# Intraspecific variation in fine-root traits is larger than in aboveground traits in European herbaceous species regardless of drought

**DOI:** 10.3389/fpls.2024.1375371

**Published:** 2024-04-09

**Authors:** Slendy Rodríguez-Alarcón, Riin Tamme, Carlos P. Carmona

**Affiliations:** Institute of Ecology and Earth Sciences, Department of Botany, University of Tartu, Tartu, Estonia

**Keywords:** analogous traits, belowground, interspecific trait variability, leaf traits, plant height, plasticity, root dry matter content, variance partitioning

## Abstract

Differences within species (Intraspecific trait variation - ITV) contribute substantially to overall trait variability and environmental harshness can reduce among-species variation. While aboveground traits have received considerable attention, knowledge about ITV in fine-root traits and how it differs from ITV in aboveground traits remains limited. This study examined the partitioning of trait variation aboveground and fine-root traits in 52 European herbaceous species and how such proportions change in response to drought, offering valuable insights for accurate functional species characterization and inter-species comparisons. We studied seven morphological aboveground and fine-root traits under drought and well-watered conditions in a greenhouse experiment. Linear mixed effect models and permutational multivariate analysis of variance (PERMANOVA) were employed to decompose trait variation, ensuring the robustness of our results. We also calculated variance partitioning for the combination of aboveground traits and the combination of fine-root traits, as well as pairs of analogous leaf and fine-root traits (i.e., traits that fulfill similar functions) for each treatment (control and drought). Among-species trait differences explained a greater proportion of overall variance than within-species variation, except for root dry matter content (RDMC). Height and leaf area stood out, with species’ identity accounting for 87-90% of total trait variation. Drought had no significant effect on the proportions of variation in any of the traits. However, the combination of fine-root traits exhibited higher intraspecific variability (44-44%) than aboveground traits (19-21%) under both drought and control. Analogous root traits also showed higher ITV (51-50%) than analogous leaf traits (27-31%). Our findings highlight substantial within-species variation and the nuanced responses of fine-root traits, particularly RDMC, suggesting root traits’ flexibility to soil heterogeneity that fosters less differentiation among species. Among-species trait differences, especially aboveground, may underscore distinct strategies and competitive abilities for resource acquisition and utilization. This study contributes to elucidate the mechanisms underlying the multifunctionality of the above- and belowground plants compartments.

## Introduction

Functional traits play a crucial role in shaping ecological processes and determining the performance of individuals, interactions within and among species, responses to environmental changes, and overall ecosystem functioning (Violle et al., 2007; Adler et al., 2014; Schmitz et al., 2015). Traditionally, trait-based ecology has primarily focused on trait differences among species (interspecific trait variation, hereafter among-species variation), however, it is becoming increasingly recognized that differences within species (intraspecific trait variation) contribute substantially to overall trait variability (Siefert et al., 2015; Westerband et al., 2021; Wong and Carmona, 2021). Consequently, intraspecific trait variation (ITV) influences shifts in species interactions, community dynamics, and ecosystem properties, exhibiting a wide variation contingent upon the species, traits, and environmental conditions (Siefert et al., 2015; Westerband et al., 2021). However, there is limited knowledge about how ITV manifests in fine-root traits and how it differs from ITV in aboveground traits. Conducting studies of this nature serves to untangle the implications of ITV both aboveground and belowground for (a) functional species characterization and (b) meaningful inter-species comparisons. (a) When traits exhibit substantial individual variation, it becomes challenging to characterize a species only based on average values, necessitating additional measurements to estimate reliable average values (Shipley et al., 2016). (b) Traits with substantial ITV prove less useful for comparison between species, particularly if this variability can be observed within the same environmental conditions. This implies that if ITV is intrinsically linked to environmental differences, such as size variations in water-available versus water-poor environments, accounting for traits of each specific environmental condition becomes imperative (Rodríguez-Alarcón et al., 2022). Therefore, investigating how the structure of trait variation proportions changes in response to environmental differences helps to unravel the ecological relevance of ITV in both above- and belowground plant compartments.

Some studies have found that the total variability for whole-plant traits, such as height, is mainly due to ITV (Luo et al., 2016; Guo et al., 2022), while others suggest a greater influence of among-species variation (de Bello et al., 2011; Dostál et al., 2020; Garcia et al., 2020). In the case of morphological leaf traits such as leaf area (LA), specific leaf area (SLA), or leaf dry matter content (LDMC), among-species variability explains most of the total variance (Luo et al., 2016; Henn et al., 2018; Firn et al., 2019; Dostál et al., 2020; Garcia et al., 2020; Streit et al., 2022; Weemstra et al., 2022). On the other hand, some studies have reported that ITV primarily drives the total variation in LA and SLA, while for LDMC the ITV is only marginally larger than among-species variation, especially within sites (Read et al., 2017; Guo et al., 2022). Regarding fine-root traits, recent studies have found that variance in specific root length (SRL) and average root diameter (AvgD) is mostly explained by among-species variation, with local differences at the same elevation exerting a modest impact (Weemstra et al., 2021, 2022). Nonetheless, ITV may also be the main contributor to the total variation in root dry matter content (RDMC) without changes with elevation (Spitzer et al., 2023). Similarly, intraspecific variability can explain most of the overall variance in SRL within sites, potentially impacting the below-ground niche breadth of a species since the differences in SRL among individuals of the same species may expand the range of ecological resources a species can use (Read et al., 2017).

Research on trait variation within and among species sheds light on the adaptive capacity of species, community assembly, ecosystem functioning, and plant performance in different environments (Laughlin et al., 2012; Jung et al., 2014; Siefert et al., 2015; Niu et al., 2020; Rodríguez-Alarcón et al., 2022). Multiple studies have investigated ITV along elevational gradients (Luo et al., 2016; Henn et al., 2018; Midolo et al., 2019; Niu et al., 2020; Weemstra et al., 2021, 2022) and environmental gradients such as soil nutrient availability, drought, and disturbances (e.g., grazing and herbivory) (de Bello et al., 2011; Jung et al., 2014; Buchmann et al., 2018; Firn et al., 2019; Guo et al., 2022; Streit et al., 2022). Certain traits, such as plant height and leaf nutrient traits, have been found to exhibit high ITV and sensibility to environmental conditions (Siefert et al., 2015; Henn et al., 2018; Guo et al., 2022) as well as some leaf morphological traits (Jung et al., 2014; Buchmann et al., 2018; Guo et al., 2022). For instance, plants growing in dry conditions exhibit slightly lower among-species variation of height compared to wet conditions, while for LDMC, intra- and interspecific variation are higher in dry sites (de Bello et al., 2011). In semiarid climates, intraspecific variation in plant height, LA, SLA, and LDMC accounts for most of the total variation in response to drought, whereas in semi-humid climates, variation in SLA and LDMC is primarily explained by species identity (Guo et al., 2022). Moreover, in dry sites, species display high intraspecific variation of SLA and SRL, but low LDMC variability within species (Weemstra et al., 2022), and the trends of intraspecific variation in root diameter vary depending on the species (Weemstra et al., 2021). Furthermore, a decline in soil moisture and nutrient availability may lead to increase in intraspecific variability for LDMC, SLA, and plant height, while simultaneously decreasing among-species variability (Niu et al., 2020). In general, these findings suggest that environmental harshness acts as a selective force that drives convergence in species traits, thereby reducing among-species variation and increasing the relative importance of intraspecific trait variation (Shipley et al., 2016; Niu et al., 2020). Exploring trait variance under harsh environmental conditions provides valuable insights into the interplay of environmental factors and species trait variation. A deeper understanding of among-species and intraspecific leaf and fine-root variability can enhance our knowledge of above- and belowground plant strategies, allowing us to better predict species responses under future drier scenarios for effective conservation and ecosystem management.

In a recent global-scale study, Carmona et al. (2021) described that the proportion of total variation explained by differences among taxonomic families is substantially larger for aboveground than for belowground traits. This result suggests that the relative importance of ITV might be larger for belowground traits, although specific studies are necessary to test this statement. Considering that prior studies on this topic have predominantly focused on a limited range of species, particularly those examining intraspecific variation in root traits (but see Read et al., 2017, 52 species), experimental studies employing a large number of species to quantify the relative contributions of intra- and interspecific variation for aboveground and fine-root traits could provide valuable insights into this issue. Moreover, compared to aboveground traits, relatively few studies have examined ITV in root traits, despite the significance of belowground ITV for optimizing resource utilization, responding to environmental changes, and influencing species interactions and ecosystem processes (Weemstra et al., 2021, 2023).

In this study, we tested seven morphological aboveground and fine-root traits: leaf area, specific leaf area, leaf dry matter content, plant height (aboveground traits), and specific root length, root dry matter content, and average root diameter (fine-root traits). These traits were measured in 52 herbaceous species under drought and well-watered conditions in a greenhouse experiment. We aimed to answer two questions: (1) What proportion of the total variability of aboveground and fine-root traits is explained by the differences among species and within species? (2) How does among-species and intraspecific trait variation change in response to drought? Since among-species variability in fine-root traits is smaller than in aboveground traits across diverse botanical families (Carmona et al., 2021), we hypothesized (H1) that the contribution of intraspecific trait variability to total variation would be higher for fine-root traits than for aboveground traits. We also predicted (H2) that the relative contribution of ITV to total trait variation would be larger under drought conditions in both aboveground and fine-root traits because the filtering effect of drought would lead to convergence in trait variation among species. As among-species trait variation decreases, the relative importance of intraspecific trait variation should increase (Shipley et al., 2016; Niu et al., 2020). Gaining a thorough comprehension of trait variation within a species in relation to environmental factors offers critical insights into how trait variation contributes to population and community resilience in the face of climate change. Understanding intraspecific trait variation also provides valuable perspectives on the underlying biological and ecological dynamics of species.

## Material and methods

### Experimental design

We established a monoculture pot experiment with 52 herbaceous species from European grassland ecosystems that cover a wide range of traits and major taxonomic families (18 families; 34 forbs, 16 graminoids, and two legumes; **Supplementary Table 1**). Seeds were obtained from Planta naturalist, a commercial supplier in Czech Republic. Pots (1L volume) were filled with a mixture of black soil (Biolan Murumuld) and sand (mix 1:1). Seeds were pre-germinated and at the end of May 2020, we transplanted seven individual seedlings of a single species per pot (one individual in the center of the pot surrounded by six individuals at about 2-3 cm distance forming a hexagon), using 10 pots for each species. Pots were randomly placed in the greenhouse of the University of Tartu, Estonia. One month later, a drought treatment was applied (5% soil volumetric water content - VWC) to half of the pots (i.e., five pots for each species), the other half were control pots (well-watered every day up to 25-28% VWC). This drought level was severe and had a substantial effect on plant biomass with reductions of up to 46.4% for some species (Rodríguez-Alarcón et al., 2022). The experiment was harvested after a month-long drought treatment (late July 2020), when the first individuals started flowering. At the end of the experiment, we had a total of 465 pots (233 in control and 232 in drought treatment; 3255 successful seedlings in total) with all seven living individuals. For more details about seed sources, germination, and treatment see Rodríguez-Alarcón et al. (2022).

### Plant functional trait measurement

We measured seven aboveground and fine-root morphological traits related to drought responses and resource use strategies. For aboveground traits, before harvesting, we measured vegetative plant height (Height, cm) and collected one young and fully expanded leaf from three individuals in each pot. For fine-root traits, we collected a sample (10-50mg) of finest roots (<2 mm) from each pot. Leaves and roots were scanned (Epson Perfection 3200 and Epson V700 photo scanner, respectively) and then dried for 72h at 60°C to measure dry leaf and root biomass. Leaf scans were processed with ImageJ software to determine leaf area (LA, mm^2^). Root scans were processed with WinRHIZO Pro 2015 (Regent Instruments Inc., Canada) to calculate average root diameter (AvgD, mm) and root length (cm). With these measurements, we estimated specific leaf area (SLA, the ratio of fresh leaf area to leaf dry mass, mm^2^ mg^-1^) and leaf dry matter content (LDMC, the ratio of leaf dry mass to leaf fresh mass, mg g^-1^) for each leaf and averaged the values for each species at the pot level. We also calculated specific root length (SRL, the ratio of root length to root dry mass, cm g^-1^) and root dry matter content (RDMC, the ratio of root dry mass to root fresh mass, mg g^-1^) for each species at the pot level, due to the complexity associated with disentangling individual roots within a pot.

### Data analysis

We estimated the source of variation for each trait by means of variance partitioning. This analysis assesses the variation in traits within-species (intraspecific trait variation) and among-species (interspecific trait variation). We first log-transformed the traits so that they fit a normal distribution, subsequently standardizing them to have a mean of 0 and standard deviation of 1. We then performed variance partitioning analysis using two different approaches: linear mixed effect models (Messier et al., 2010; Carmona et al., 2015) and permutational multivariate analysis of variance (PERMANOVA) (Carmona et al., 2021; de Bello et al., 2021).

In the case of linear mixed models, we fitted separate models for every trait and treatment (control and drought), including species as a random factor (random ~ 1 | Species). We also fitted models considering simultaneously species and treatment as random factors (random ~ 1 | Treatment, ~ 1 | Species). -Models using species nested in treatment (random ~ 1 | Treatment/Species) yielded similar results (not shown)-. This approach allowed us to decompose trait variation (i.e., how much of total trait variation is due to the considered random factors) for each aboveground (LA, SLA, LDMC, height) and fine-root (AvgD, SRL, RDMC) trait. We built these models using the R function “lme” from nlme package (Pinheiro et al., 2023) and calculated variance partitioning with the “varcomp” function from ape package (Paradis et al., 2023). We also ran the same set of analyses separately for graminoids (grasses) and forbs.

In the case of the permutational test, we first estimated the dissimilarities between all pairs of species using Euclidean distances based on the scaled traits. We created dissimilarity matrices considering each single trait, as well as the combination of aboveground traits, and the combination of fine-root traits, both for each treatment separately (control and drought) and for treatments together. Likewise, to have more fair comparison of ITV above- and belowground, we used analogous traits of leaves and fine-roots that serve similar functions in resource acquisition strategies (Reich, 2014; Weemstra et al., 2022). For this, we created dissimilarity matrices using aboveground traits that have a clear corresponding analogous fine-root traits (SLA and LDMC) and vice versa (SRL and RDMC). We then analyze these dissimilarity matrices using PERMANOVA with the “adonis” function from vegan package (Oksanen et al., 2022). In the case of dissimilarity matrices from single treatments, we made a PERMANOVA for each treatment (one for control and one for drought), using species as the explanatory variable. In the case of the dissimilarity matrices based on both treatments together, we used species, treatment, and the species*treatment interaction as explanatory variables. This approach allows us to estimate how much of the total trait variation is due to differences among the different predictors (i.e., differences among species and among treatments). To evaluate if there is any differentiated response of ITV between grasses and forbs, we ran the same set of analyses considering independently each of these growth forms. Due to the limited number of legume species in our data set, we opted not to conduct a separate analysis for legumes.

We compared the outcomes of both model approaches to confirm the consistency of the results regarding both interspecific and intraspecific variability of aboveground and fine-root traits and whether drought generated any changes in these proportions. All data analyses were performed with R version 4.2.0 (R Foundation for Statistical Computing, Vienna, AT).

## Results

**Figure 1:**
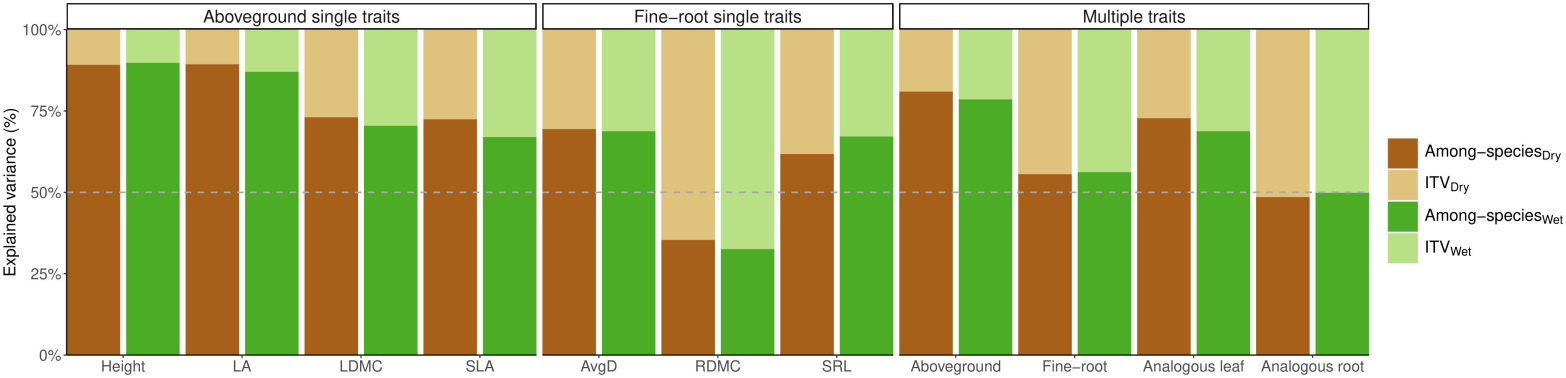
Variance partitioning of PERMANOVAs for each individual trait (single traits), and multi-traits: for the combination of aboveground traits (LA, SLA, LDMC, and Height), the combination of fine-root traits (AvgD, SRL, and RDMC), analogous leaf traits (SLA, LDMC), and analogous root traits (SRL, RDMC), considering each treatment separately: control (green bars) and drought (brown bars). In all cases, the sum of intraspecific trait variation (ITV) and interspecific trait variation (among-species) add up to 100% of the total variation. Log-traits were considered. LA, leaf area; SLA, specific leaf area; LDMC, leaf dry matter content; SRL, specific root length; AvgD, average root diameter; RDMC, root dry matter content.

Differences among-species explained most of the variation in aboveground and fine-root traits, except for root dry matter content (RDMC) where the total variation was mostly attributed to intraspecific trait variation. Two aboveground traits, height and leaf area (LA) showed the highest proportion of variance explained by species identity (**Figure 1**). The proportion of variation due to ITV in the remaining aboveground (leaf dry matter content and specific leaf area) and fine-root traits (average root diameter and specific root length) was similar (ranging between 67-73% for aboveground traits and 62-69% for fine-root traits). When all fine-root traits were considered together, the proportion of variation due to ITV was only slightly smaller than the proportion due to differences among species. Similarly, ITV of analogous root traits explained half of the total variance under drought (51%) and control (50%). In contrast, the differences among species surpassed that of ITV by over threefold for combined aboveground traits and more than twofold for analogous leaf traits (**Figure 1**). On average, the contribution of within-species variation (ITV) to the total variation was 2.2 times higher in belowground traits compared to aboveground traits (44% on average for fine-root traits vs. 20% for aboveground traits), and 1.76 higher for analogous traits in roots than leaves (51% on average for analogous root traits vs. 29% for analogous leaf traits). Similarly, when considering the combination of all aboveground traits, ITV explained only a small proportion of overall variation (21% in control and 19% under drought), and ITV of analogous leaf traits accounted for 31% of variation in control and 27% under drought conditions. These results between the linear mixed effects model and the permutational multivariate analysis of variance (PERMANOVA) were highly consistent (r = 0.99, p < 0.01 for ITV both drought and control treatments separately). Because PERMANOVA analysis allows us to combine multiple traits, we present the results of these models in the main text. The results of the linear mixed effects models for both treatments are presented in the (**Supplementary Table 2**).

Across traits, combined fine-root and aboveground traits along with the analogous leaf traits, the variation among species consistently had the highest contribution. However, it is noteworthy that analogous leaf traits accounted for fifty percent of the total variance. The drought treatment had minimal effects on all evaluated traits (max R^2^ = 0.04), despite being statistically significant for most traits, except for height and LA. Similarly, the species and treatment interaction displayed lack of significance for most traits with notably low R^2^ values (max R^2^ = 0.08), showing that despite the consistent response across different species, the effect of the treatment on these responses is negligible (**Table 1**). The corresponding results of the linear mixed effects models for each trait are shown in (**Supplementary Table 3**), which were highly consistent with the results from PERMANOVAs for the proportion of variance explained by differences within species (r = 0.99, p < 0.01).

**Table 1:** PERMANOVAs for each trait, for the combination of aboveground traits, and for the combination of fine-root traits, considering species and treatment interaction.

	Source of variation	Df	SumOfSqs	R2	F	p-value
Aboveground traits						
logHeight	Among Species	51	400.7	0.871	58.5214	**0.001**
	Treatment	1	0.36	0.001	2.6658	0.105
	Sp*Treatment	51	11.01	0.024	1.6079	**0.007**
	Within Species	357	47.93	0.104		
logLA	Among Species	51	397.48	0.864	51.0712	**0.001**
	Treatment	1	0.44	0.001	2.8637	0.091
	Sp*Treatment	51	7.61	0.017	0.9774	0.53
	Within Species	357	54.48	0.118		
logLDMC	Among Species	51	318.12	0.692	17.2856	**0.001**
	Treatment	1	2.46	0.005	6.821	**0.008**
	Sp*Treatment	51	10.6	0.023	0.5759	0.992
	Within Species	357	128.82	0.280		
logSLA	Among Species	51	280.12	0.609	14.611	**0.001**
	Treatment	1	18.76	0.041	49.902	**0.001**
	Sp*Treatment	51	26.92	0.059	1.404	0.059
	Within Species	357	134.2	0.292		
Above traits	Among Species	51	1396.41	0.759	26.7486	**0.001**
	Treatment	1	22.02	0.012	21.5071	**0.001**
	Sp*Treatment	51	56.13	0.031	1.0752	0.266
	Within Species	357	365.44	0.199		
Fine-root traits						
logAvgD	Among Species	51	293.23	0.637	14.6347	**0.001**
	Treatment	1	6.24	0.014	15.872	**0.001**
	Sp*Treatment	51	20.28	0.044	1.0121	0.455
	Within Species	357	140.26	0.305		
logRDMC	Among Species	51	293.23	0.276	14.6347	**0.001**
	Treatment	1	6.24	0.011	15.872	**0.001**
	Sp*Treatment	51	20.28	0.057	1.0121	0.455
	Within Species	357	140.26	0.656		
logSRL	Among Species	51	251.94	0.548	11.1392	**0.001**
	Treatment	1	11.61	0.025	26.1798	**0.001**
	Sp*Treatment	51	38.13	0.083	1.6856	**0.004**
	Within Species	357	158.32	0.344		
Below traits	Among Species	51	671.97	0.487	7.8328	**0.001**
	Treatment	1	22.71	0.016	13.5007	**0.001**
	Sp*Treatment	51	84.79	0.061	0.9884	0.517
	Within Species	357	600.52	0.435		
Analogous traits						
Leaf traits	Among Species	51	598.24	0.650	15.921	**0.001**
	Treatment	1	21.22	0.023	28.8019	**0.001**
	Sp*Treatment	51	37.52	0.041	0.9985	0.496
	Within Species	357	263.03	0.286		
Root traits	Among Species	51	378.74	0.412	5.7601	**0.001**
	Treatment	1	16.47	0.018	12.7781	**0.001**
	Sp*Treatment	51	64.52	0.070	0.9812	0.547
	Within Species	357	460.27	0.500		

Above is the combination of traits: LA, SLA, LDMC, height. Below is the combination of traits: AvgD, SRL, RDMC. Leaf are the analogous traits: SLA, LDMC. Root are the analogous traits: SRL, RDMC. Significant p-values are shown in bold text (p<0.05). Log-traits were considered. LA, leaf area; SLA, specific leaf area; LDMC, leaf dry matter content; SRL, specific root length; AvgD, average root diameter; RDMC, root dry matter content.

**Figure 2:**
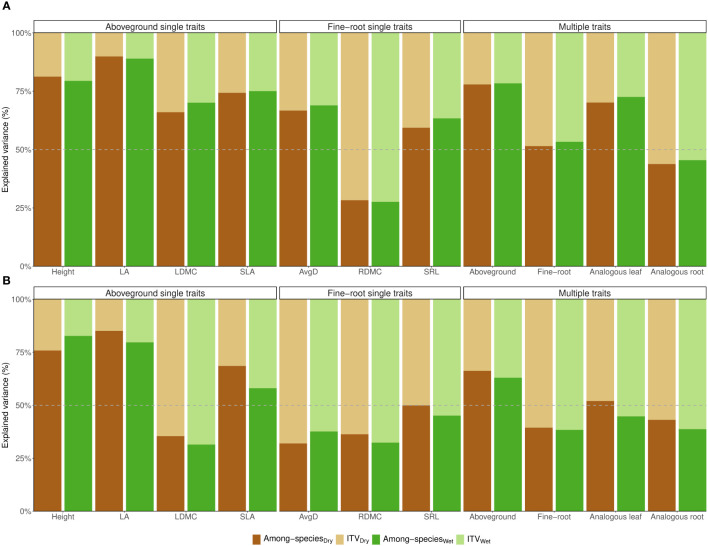
Variance partitioning of PERMANOVAs for **(A)** graminoids and **(B)** forbs separately. Models are for each individual trait (single traits), and multi-traits: for the combination of aboveground traits (LA, SLA, LDMC, and Height), the combination of fine-root traits (AvgD, SRL, and RDMC), analogous leaf traits (SLA, LDMC), and analogous root traits (SRL, RDMC), considering each treatment separately: control (green bars) and drought (brown bars). In all cases, the sum of intraspecific trait variation (ITV) and interspecific trait variation (among-species) add up to 100% of the total variation. Log-traits were considered. Traits abbreviations are explained in **Figure 1**.

When graminoids and forbs were analyzed separately, ITV predominantly accounted for the variation in RDMC of forbs (**Figure 2A**). In the case of grasses, intraspecific variation was higher for LDMC, AvgD, and RDMC in both treatments, as well as for the combination of fine-root traits and analogous root traits. Notably, for SRL and analogous leaf traits, intraspecific differences explained half or slightly more than half of the total variance (**Figure 2B**). On average, the contribution of ITV was 1.96 times higher for analogous root traits in forbs (55% vs. 28% on average) and 1.15 times higher in grasses (59% vs. 51% on average) (**Figure 2**). When examining the combination of all aboveground traits, within-species variation (ITV) explained 37% in control and 34% under drought for grasses, and 21% in control and 22% under drought for forbs. Likewise, ITV of analogous leaf traits accounted for 27% in control and 30% under drought for forbs. However, within-species variation explained slightly more than half of the total variation in analogous leaf traits for grasses (51% on average) (**Figure 2B**). The drought treatment had minimal effects on all evaluated traits (max R^2^ = 0.1 in grasses; max R^2^ = 0.07 in forbs) and the interaction between species and treatment was not significant for either growth form (max R^2^ = 0.06 in grasses; max R^2^ = 0.09 in forbs) (**Supplementary Tables 4**, **5**). These results were highly consistent with the corresponding linear mixed effects models (**Supplementary Tables 6**, **7**).

## Discussion

We explored how the partitioning of trait variation in among-species and intraspecific components differ between aboveground and fine-root traits, and to what extent this variation is also explained by water availability in a set of 52 species typical for European grasslands. We found that among-species trait variation explains a larger proportion of variance than ITV, except in the case of root dry matter content (RDMC), for which ITV accounted for twice as much variation as among-species differences. Interestingly, the total variability in leaf dry matter content (LDMC) and average root diameter (AvgD) was also primarily reflected by intraspecific variation when considering graminoids separately. Moreover, our results show that the proportion of total variation associated with reduced water availability was very small for all traits and trait combinations, despite the substantial reductions in plant biomass for most species under drought conditions (**Supplementary Figure 1**) (Rodríguez-Alarcón et al., 2022). These results suggest that while trait-based approaches remain reliable in comparing species, the extent of variation due to differences among conspecifics depends on the specific trait under consideration. This can be particularly important for less-explored fine-root traits such as RDMC, highlighting that differences in the partitioning of trait variance cannot be reliably attributed to differences among-species.

We found support for our H1 since the proportion of total variation due to ITV was 2.2 times higher belowground than aboveground. Similarly, the contribution of ITV was 1.76 times higher for analogous traits in roots than leaves (**Figure 1**). This trend persisted when analyzing grasses and forbs separately, with the contribution of ITV 1.96 times higher for root traits compared to analogous leaf traits in forbs and 1.15 times higher in grasses (**Figure 2**). Exploration of ITV in fine-root traits has been limited so far (Siefert et al., 2015). Spitzer et al. (2023) and Read et al. (2017) found that ITV was larger than differences among species for RDMC and SRL, respectively. Furthermore, when considering analogous traits, Weemstra et al. (2022) reported that intraspecific variation was greater in root traits compared to leaf traits. Higher ITV in fine-root traits compared to aboveground traits could be due to the heterogeneity in soil conditions and microbial interactions that promote a greater variation within species as individuals uniquely adapt to their immediate soil environment (Paganeli and Batalha, 2021; Weemstra et al., 2022; Spitzer et al., 2023). This also leads to a stronger below- than aboveground competition among conspecifics, arising from differential responses to the heterogeneous belowground conditions over time (Read et al., 2017). Moreover, the mycorrhizal colonization rate of a species could change with the root architecture of the individuals (Bergmann et al., 2020), and it is known that the mycorrhizal types and statuses influence plants’ niche differentiation and expansion (Gerz et al., 2018). All these factors might lead to more flexibility for allocating resources belowground which enhances the adaptability of a species to a wider range of resource availability and environmental conditions, and promotes higher intraspecific variability. As a consequence, fine-root traits may exhibit a more nuanced response that leads to less trait differentiation among species, as previously shown at the family level (Carmona et al., 2021; Bueno et al., 2023). In contrast, aboveground traits may be subject to stronger evolutionary constraints, leading to more pronounced differences among species and families (Carmona et al., 2021; Tumber-Dávila et al., 2022; Capdevila et al., 2023). Finally, the significant contribution of within-species variability that we found when combining fine-root traits might imply a wider belowground niche of a species due to differences among its individuals. However, the use of ITV to assess niche breadth should be explored in roots just as it has been explored in some aboveground traits (Gerz et al., 2018; Fajardo and Siefert, 2019; Treurnicht et al., 2020; Bergholz et al., 2021). Our results also suggest that considering multiple traits from different individuals within a species might be more reliable than considering only mean trait values for functional species characterization and for understanding the importance of trait sets in shaping a plant’s phenotype, particularly belowground (Weemstra et al., 2021; Westerband et al., 2021; Wong and Carmona, 2021). This perspective may have implications for trait space occupation, plant strategies, and ecosystem multifunctionality, particularly in soils (Messier et al., 2017; Hanisch et al., 2020; Rodríguez-Alarcón et al., 2022).

Results from variance partitioning analysis indicated that the among-species differences explained more than 60% of the total variation for all aboveground traits and analogous leaf traits if all growth forms were analyzed together. Height and leaf area were particularly prominent in this sense since species’ identity explained between 87%-90% of their total variation for all growth forms (**Figure 1**), 79%-82% for grasses, and 80%-89% for forbs (**Figure 2**). Consistent with Bueno et al. (2023)’s findings, our observations also underscore that the prominent source of height variation resides between families (75% in control and 68% in dry conditions), confirming that plant height is a highly conserved trait (Tumber-Dávila et al., 2022), even in a dataset that contains only herbaceous species. Low proportions of ITV in plant height and leaf area, as observed by Siefert et al. (2015) (~26% and ~16%, respectively), suggest limited plasticity in size-related traits that may be due to genetic restrictions or trade-off constraints within individuals but that does not necessarily imply lack of adaptation to environmental gradients (Palacio-López et al., 2015; Siefert et al., 2015). Our results for both traits reflect that differences in plant size are mainly species-specific, which emphasizes that different plant species have evolved distinct strategies and competitive abilities, particularly in terms of light acquisition (Vogel et al., 2019; Meilhac et al., 2020; Bueno et al., 2023). Likewise, for all growth forms, differences among species explained between 67%-72% and 70%-73% of the variation in SLA and LDMC under control and drought treatment respectively (**Figure 1**). The wide range of values of these traits among species suggests a diversity of strategies for resource acquisition and utilization that in turn allow a variety of responses under changing conditions (Reich, 2014; Firn et al., 2019; Weemstra et al., 2022). A diverse array of these morphological traits among species can help maintain different species’ roles and strategies linked to the leaf economics spectrum, which in turn underlie ecosystem-level processes such as nutrient cycling, decomposition, and productivity (Reich, 2014; Szefer et al., 2017). Similarly, when considering the combination of all aboveground traits, ITV explained only a small proportion of overall variation (**Figures 1**, **2**). Different challenges and opportunities that plants may face aboveground, for example, competition for light, herbivory, and fire dynamics, could lead to a greater functional differentiation among different species to cope with these pressures (Carmona et al., 2021). The large variation of aboveground trait values among species might facilitate species coexistence by differentiation of resource use strategies allowing for niche differentiation (Meilhac et al., 2020; Streit et al., 2022). However, in some cases it is still imperative to have data on ITV at the community level to understand the stability and dynamics of plant communities (Westerband et al., 2021; Wong and Carmona, 2021; Streit et al., 2022). Furthermore, the large proportion explained by ITV in LDMC for grasses (66% on average) enhances the significance of ITV in analogous leaf traits (51% on average) (**Figure 2B**) and suggest flexibility in growth rates, herbivory resistance, and plant economics, which is particularly useful in grassland ecosystems that may experience different disturbance regimes as grazing, fire, and land use (Gross et al., 2007; Blumenthal et al., 2020).

For fine-root traits, information about the amount of variation among and within species is scarce, particularly for root dry matter content (Herz et al., 2017; Spitzer et al., 2023). In our dataset, RDMC was the trait with the largest proportion explained by within-species variation with no strong or significant changes due to drought for all growth forms (67% and 65% under control and drought respectively) and separately for grasses (68% in control and 64% under drought) and forbs (72% under control and drought). Similar results were reported by Spitzer et al. (2023) who found that within-species variation in this trait remains unaffected by shifting environmental conditions along an elevation gradient. Yet, intraspecific trait variation in RDMC can be affected by both local neighborhood diversity and soil erosion caused by land-use intensity (Herz et al., 2017). This underscores the importance of intraspecific root trait variation for a species in adapting to changing environments, particularly in the context of root-soil interactions (Spitzer et al., 2023). Additionally, high levels of ITV in RDMC suggest that there is no uniform strategy within species, providing plant population with adaptability to thrive in diverse soil conditions by adjusting their resource allocation strategies, either in scenarios of optimal water availability or drought. As RDMC is a surrogate of fine root tissue density (Birouste et al., 2014), it is likely that some individuals prioritize higher dry matter allocation to their fine roots, potentially obtaining longer-lived roots. Conversely, other individuals may emphasize nitrogen uptake for fast resource investment, albeit with a shorter lifespan (Bergmann et al., 2020; Carmona et al., 2021). This trait flexibility endows the species with a wide range of strategies for acquisition and storage of nutrients and soil water that allows it to cope with fluctuating conditions faster and more effectively, which would help increase its resilience (Zwicke et al., 2015; Rodríguez-Alarcón et al., 2022; Spitzer et al., 2023). The fact that the proportion of trait variation due to differences between water treatments was also very low for this trait seems to support this explanation. In the case of SRL and AvgD, we found lower variability within species for all growth forms, explaining only 33%-38% and 31%-30% under well-watered and drought conditions respectively (**Figure 1**). Our findings are in line with some previous studies where differences among-species were the main component of the overall variance in these root traits, which suggests that the plant responses to different environments depend on the species patterns to modify resource acquisition and conservation, highlighting the multidimensionality of the belowground phenotype (Bergmann et al., 2020; Carmona et al., 2021; Weemstra et al., 2021, 2023). Additionally, these traits seem versatile in graminoids, where intraspecific variation drove most of the total variation in SRL and AvgD, explaining 55%-50% and 62%-68% under well-watered and drought conditions respectively (**Figure 2B**). Considerable intraspecific variation in specific root length (SRL) might enable diverse nutrient absorption capacities and the deployment of strategies for particular competitive advantages in heterogeneous soils, especially with patchy nutrient distribution (Ravenek et al., 2016). The substantial within-species variation observed not only in specific root length (SRL) but also in average root diameter (AvgD) underlines the adaptability of grasses to optimize resource uptake across diverse soil conditions. These findings implicate a broad spectrum of strategies within the root economic spectrum, encompassing efficient soil exploration with cost-effective roots to collaborative resource acquisition through mycorrhizal fungi (Bergmann et al., 2020).

Contrary to our expectations (H2), we found a negligible effect of water availability on trait variation. While shifts towards more intraspecific trait variation have been observed with decreasing water availability (Guo et al., 2022; Weemstra et al., 2022), our results indicated that drought did not significantly change the proportion of variation within species. However, our species declined in biomass under drought conditions (Rodríguez-Alarcón et al., 2022), which suggests that stabilizing trait variation could be a strategy to maintain core physiological functions while plants adjust biomass to cope with water limitation. Our findings may reflect that the ability of species to respond to different water-availability conditions is maintained since the proportion of trait variation is relatively stable within and among species even under drought conditions, which is important for plant community stability (Herz et al., 2017; Hetzer et al., 2021; Luo et al., 2023). Given that herbaceous species in temperate climate have developed adaptative responses to cope with seasonal stresses ranging from freezing to drought (Gillespie and Volaire, 2017), it may be worth exploring if persistence in the proportions of trait variation remains across a spectrum of humid habitat species subjected to drought. Likewise, the absence of intraspecific changes in response to drought treatment might suggest that trait differences at the community level are more likely attributed to shifts in community composition (species’ turnover) rather than intraspecific adaptations, but this potential outcome requires verification (Weemstra et al., 2021; Streit et al., 2022). However, finding no changes in aboveground and fine-root morphological traits in response to drought does not exclude changes in other traits. Subsequent investigations could explore additional traits, such as leaf and root nutrient concentration, which are anticipated to exhibit higher ITV than morphological traits in response to site-specific conditions like resource availability (Siefert et al., 2015; Firn et al., 2019). For example, traits such as leaf N:P ratio, leaf K, leaf P, root N:P ratio, root N, and root C:P ratio exhibited considerable variation across environmental conditions (Siefert et al., 2015; Spitzer et al., 2023), providing potential areas for further ITV exploration. Likewise, it is possible that other traits more directly related to the performance of individuals, such as seed size that determines regeneration and seedling performance, may display distinct responses to drought, especially under natural conditions (Martínez-López et al., 2020). Assessing traits in natural settings becomes inherently more challenging due to the dynamic and unpredictable nature of ecosystems. Factors such as variations in soil composition, microclimate, and interspecific interactions can significantly affect trait variation and plant responses to drought. While our study provides valuable information on ITV in response to drought in a controlled environment, we recognize the need for further research to explore trait variation and its implications for coping with drought under more realistic field conditions. Moreover, plasticity in response to drought might also be influenced by other factors that were not considered in our study, such as mycorrhizal symbiosis (Puy et al., 2022; Bueno et al., 2022) and soil microbiome diversity (bacteria, viruses, and protists) that can enhance physiological and biochemical plant strategies to mitigate drought stress (Valencia et al., 2018; Poudel et al., 2021).

## Conclusion

Overall, our findings corroborate previous results showing that most of the total trait variance is explained by among-species differences for most of the analyzed single traits, both aboveground (Dostál et al., 2020; Garcia et al., 2020; Streit et al., 2022; Weemstra et al., 2022) and belowground (Weemstra et al., 2021, 2022). The fact that traits display high among-species variation can lead to complementarity in resource use, ecosystem stability, and diverse responses to environmental changes so that species can persist and coexist. Yet, the major proportion of trait variation is not invariably ascribed to species distinctions since it depends on the pool of species considered, the trait of interest and the growth form (Weemstra et al., 2021; Streit et al., 2022). For example, ITV was the most important component of the overall variation in RDMC, which suggests a wider range of abilities of a species to access and store resources from the soil. Furthermore, nearly half of the variance in both combined fine-root traits and analogous root traits comes from intraspecific differences, underscoring the importance of studying trait sets from different individuals within a species for accurate functional species characterization belowground. Additionally, our results suggest that, at least for this pool of species, drought response is not associated with substantial changes in the proportion of trait variation that is explained by within-species differences. However, since our current assessment relies only on experimental observations, it is essential to acknowledge that within-species variation may display greater variability in natural environments (Carmona et al., 2015; Firn et al., 2019; Dostál et al., 2020; Wong and Carmona, 2021). Moreover, delving into the origins of ITV would enhance our comprehension of the complex dynamics shaping plant traits, as ITV arises from either genotypic variation within a population or the variation of trait values within genotypes (de Bello et al., 2021; Puy et al., 2021b). Future studies should disentangle the role of genetic diversity on intraspecific phenotypic variation and consider nutrient leaf and root traits as well as different growth forms (Puy et al., 2021a; Streit et al., 2022; Weemstra et al., 2022). This information is essential to disentangle the interplay of inter- and intraspecific trait variation in species resilience and community dynamics, shedding light on the nuanced above- and belowground responses of plants to environmental shifts.

## Data availability statement

The original dataset for this study is available from Dryad https://doi.org/10.5061/dryad.vdncjsxxk (Rodriguez Alarcon et al., 2022). List of species is included in the Supplementary Material.

## Author contributions

SR: Data curation, Formal analysis, Investigation, Methodology, Writing – original draft, Writing – review & editing. RT: Supervision, Writing – review & editing. CC: Investigation, Methodology, Resources, Supervision, Writing – review & editing.
