# Supplementary material for: Intraspecific variation in fine-root traits is larger than in aboveground traits in European herbaceous species regardless of drought
Source: Front Plant Sci. 2024 Apr 9;15:1375371. doi: 10.3389/fpls.2024.1375371 (PMC11035731; doi:10.3389/fpls.2024.1375371)
Supplement: Supplementary file 1 [file DataSheet_1.docx]

Supplementary Material

**Supplementary Table 1**. List of 52 species included in the study with information about family, growth habit, photosynthetic pathway, mycorrhizal symbiosis type, and mycorrhizal status for each species. AM: Arbuscular mycorrhizal symbiosis type; OM: Obligately mycorrhizal; FM: Facultatively mycorrhizal.

| Species | Family | Growth habit | Photosynthetic pathway | Plant mycorrhizal type | Plant mycorrhizal status |
| --- | --- | --- | --- | --- | --- |
| *Achillea millefolium* | Asteraceae | Forb | C3 | AM | FM |
| *Agrostis capillaris* | Poaceae | Graminoid | C3 | AM | FM |
| *Agrostis gigantea* | Poaceae | Graminoid | C3 | AM | OM |
| *Agrostis stolonifera* | Poaceae | Graminoid | C3 | AM | FM |
| *Alopecurus pratensis* | Poaceae | Graminoid | C3 | AM | OM |
| *Anthoxanthum odoratum* | Poaceae | Graminoid | C3 | AM | FM |
| *Artemisia campestris* | Asteraceae | Forb | C3 | AM | FM |
| *Briza media* | Poaceae | Graminoid | C3 | AM | FM |
| *Campanula persicifolia* | Campanulaceae | Forb | C3 | AM | FM |
| *Campanula rapunculoides* | Campanulaceae | Forb | C3 | AM | OM |
| *Cirsium vulgare* | Asteraceae | Forb | C3 | AM | FM |
| *Dactylis glomerata* | Poaceae | Graminoid | C3 | AM | FM |
| *Daucus carota* | Apiaceae | Forb | C3 | AM | FM |
| *Deschampsia cespitosa* | Poaceae | Graminoid | C3 | AM | FM |
| *Epilobium angustifolium* | Onagraceae | Forb | C3 | AM | FM |
| *Festuca pratensis* | Poaceae | Graminoid | C3 | AM | FM |
| *Festuca rubra* | Poaceae | Graminoid | C3 | AM | FM |
| *Fragaria vesca* | Rosaceae | Forb | C3 | AM | FM |
| *Galium album* | Rubiaceae | Forb | C3 | AM | OM |
| *Hieracium umbellatum* | Asteraceae | Forb | C3 | AM | FM |
| *Hypericum maculatum* | Hypericaceae | Forb | C3 | AM | OM |
| *Hypericum perforatum* | Hypericaceae | Forb | C3 | AM | FM |
| *Leontodon autumnalis* | Asteraceae | Forb | C3 | AM | OM |
| *Leontodon hispidus* | Asteraceae | Forb | C3 | AM | OM |
| *Linaria vulgaris* | Scrophulariaceae | Forb | C3 | AM | FM |
| *Lotus corniculatus* | Fabaceae | Legume | C3 | AM | FM |
| *Lychnis flos-cuculi* | Caryophyllaceae | Forb | C3 | AM | FM |
| *Lysimachia vulgaris* | Primulaceae | Forb | C3 | AM | FM |
| *Phleum pratense* | Poaceae | Graminoid | C3 | AM | FM |
| *Plantago lanceolata* | Plantaginaceae | Forb | C3 | AM | FM |
| *Plantago media* | Plantaginaceae | Forb | C3 | AM | OM |
| *Poa compressa* | Poaceae | Graminoid | C3 | AM | FM |
| *Poa nemoralis* | Poaceae | Graminoid | C3 | AM | FM |
| *Poa palustris* | Poaceae | Graminoid | C3 | AM | FM |
| *Poa pratensis* | Poaceae | Graminoid | C3 | AM | FM |
| *Poa trivialis* | Poaceae | Graminoid | C3 | AM | FM |
| *Potentilla argentea* | Rosaceae | Forb | C3 | AM | OM |
| *Prunella vulgaris* | Lamiaceae | Forb | C3 | AM | FM |
| *Ranunculus acris* | Ranunculaceae | Forb | C3 | AM | OM |
| *Rumex acetosa* | Polygonaceae | Forb | C3 | AM | FM |
| *Rumex acetosella* | Polygonaceae | Forb | C3 | AM | FM |
| *Silene nutans* | Caryophyllaceae | Forb | C3 | AM | FM |
| *Solidago canadensis* | Asteraceae | Forb | C3 | AM | FM |
| *Solidago virgaurea* | Asteraceae | Forb | C3 | AM | OM |
| *Tanacetum vulgare* | Asteraceae | Forb | C3 | AM | FM |
| *Trifolium repens* | Fabaceae | Legume | C3 | AM | FM |
| *Urtica dioica* | Urticaceae | Forb | C3 | AM | FM |
| *Valeriana officinalis* | Valerianaceae | Forb | C3 | AM | FM |
| *Verbascum nigrum* | Scrophulariaceae | Forb | C3 | AM | OM |
| *Verbascum thapsus* | Scrophulariaceae | Forb | C3 | AM | OM |
| *Veronica officinalis* | Scrophulariaceae | Forb | C3 | AM | FM |
| *Veronica teucrium* | Scrophulariaceae | Forb | C3 | AM | OM |

*Leontodon autumnalis* is synonym of *Scorzoneroides autumnalis*; *Lychnis flos-cuculi* is synonym of *Silene flos-cuculi*; *Veronica teucrium* is synonym of *Veronica austriaca*. Information about mycorrhizal symbiosis type and mycorrhizal status for each species is from Bueno et al., 2017, 2021.

**Supplementary Table 2**. Linear mixed effects models for both treatments considering species as a random factor. Proportion of variance explained by species and within species. Log-traits were considered. Height (H, cm), Leaf Area (LA, mm^2^), Specific Leaf Area (SLA, mm^2^ mg^-1^), Leaf Dry Matter Content (LDMC, mg g^-1^), Specific Root Length (SRL, cm g^-1^), Average root Diameter (AvgD, mm), and Root Dry Matter Content (RDMC, mg g^-1^).

| **Treatment** | **Trait** | **Species** | **Within Species** |
| --- | --- | --- | --- |
| Wet | Height | 0.884 | 0.116 |
|  | LA | 0.835 | 0.165 |
|  | LDMC | 0.623 | 0.377 |
|  | SLA | 0.573 | 0.427 |
|  | AvgD | 0.627 | 0.373 |
|  | RDMC | 0.142 | 0.858 |
|  | SRL | 0.595 | 0.405 |
| Dry | Height | 0.878 | 0.122 |
|  | LA | 0.860 | 0.140 |
|  | LDMC | 0.659 | 0.341 |
|  | SLA | 0.639 | 0.361 |
|  | AvgD | 0.616 | 0.384 |
|  | RDMC | 0.176 | 0.824 |
|  | SRL | 0.505 | 0.495 |

**Supplementary Table 3**. Linear mixed effects models for each single trait considering species and treatment as random factors. Proportion of variance explained by species, treatment, and within species. Log-traits were considered. Traits abbreviations are explained in Supplementary Table 2.

| **Trait** | **Species** | **Treatment** | **Within Species** |
| --- | --- | --- | --- |
| Height | 0.880 | 3.47E-09 | 0.120 |
| LA | 0.845 | 6.49E-09 | 0.155 |
| LDMC | 0.641 | 6.22E-09 | 0.359 |
| SLA | 0.557 | 7.69E-02 | 0.366 |
| SRL | 0.540 | 2.32E-02 | 0.437 |
| AvgD | 0.619 | 4.35E-03 | 0.376 |
| RDMC | 0.152 | 1.52E-02 | 0.833 |

**Supplementary Table 4**. PERMANOVAs considering only forbs. Models for each trait, for the combination of aboveground traits, and for the combination of fine-root traits, considering species and treatment interaction. *Above* is the combination of traits: LA, SLA, LDMC, height. *Below* is the combination of traits: AvgD, SRL, RDMC. Analogous leaf and fine-root trait pairs: SLA, LDMC (*Leaf*), SRL, RDMC (*Root*). Significant p-values are shown in bold text (p<0.05). Trait abbreviations are explained in Supplementary Table 2.

|  |  | **Source of variation** | **Df** | **SumOfSqs** | **R2** | **F** | **p-value** |
| --- | --- | --- | --- | --- | --- | --- | --- |
| **Aboveground traits** | |  |  |  |  |  |  |
| logHeight | | Among Species | 33 | 221.319 | 0.763 | 26.1386 | **0.001** |
|  | | Treatment | 1 | 0.147 | 0.001 | 0.5727 | 0.423 |
|  | | Sp*Treatment | 33 | 11.317 | 0.039 | 1.3366 | 0.119 |
|  | | Within Species | 223 | 57.217 | 0.197 |  |  |
|  | |  |  |  |  |  |  |
| logLA | | Among Species | 33 | 253.841 | 0.875 | 56.1037 | **0.001** |
|  | | Treatment | 1 | 1.075 | 0.004 | 7.8373 | **0.008** |
|  | | Sp*Treatment | 33 | 4.51 | 0.016 | 0.9967 | 0.494 |
|  | | Within Species | 223 | 30.575 | 0.105 |  |  |
|  | |  |  |  |  |  |  |
| logLDMC | | Among Species | 33 | 183.267 | 0.632 | 13.5792 | **0.001** |
|  | | Treatment | 1 | 6.241 | 0.022 | 15.2592 | **0.001** |
|  | | Sp*Treatment | 33 | 9.292 | 0.032 | 0.6885 | 0.903 |
|  | | Within Species | 223 | 91.201 | 0.314 |  |  |
|  | |  |  |  |  |  |  |
| logSLA | | Among Species | 33 | 178.593 | 0.616 | 17.7667 | **0.001** |
|  | | Treatment | 1 | 20.652 | 0.071 | 67.797 | **0.001** |
|  | | Sp*Treatment | 33 | 22.827 | 0.079 | 2.2709 | **0.002** |
|  | | Within Species | 223 | 67.928 | 0.234 |  |  |
|  | |  |  |  |  |  |  |
| Above traits | | Among Species | 33 | 837.02 | 0.722 | 22.907 | **0.001** |
|  | | Treatment | 1 | 28.11 | 0.024 | 25.3902 | **0.001** |
|  | | Sp*Treatment | 33 | 47.95 | 0.041 | 1.3121 | **0.023** |
|  | | Within Species | 223 | 246.92 | 0.213 |  |  |
| **Fine-root traits** | |  |  |  |  |  |  |
| logAvgD | | Among Species | 33 | 179.094 | 0.618 | 13.1193 | **0.001** |
|  | | Treatment | 1 | 2.705 | 0.009 | 6.5387 | **0.012** |
|  | | Sp*Treatment | 33 | 15.952 | 0.055 | 1.1685 | 0.238 |
|  | | Within Species | 223 | 92.249 | 0.318 |  |  |
|  | |  |  |  |  |  |  |
| logRDMC | | Among Species | 33 | 59.743 | 0.206 | 1.965 | **0.006** |
|  | | Treatment | 1 | 5.207 | 0.018 | 5.6513 | **0.02** |
|  | | Sp*Treatment | 33 | 19.591 | 0.068 | 0.6443 | 0.93 |
|  | | Within Species | 223 | 205.459 | 0.708 |  |  |
|  | |  |  |  |  |  |  |
| logSRL | | Among Species | 33 | 150.488 | 0.519 | 9.1457 | **0.001** |
|  | | Treatment | 1 | 1.516 | 0.005 | 3.0399 | 0.07 |
|  | | Sp*Treatment | 33 | 26.804 | 0.092 | 1.629 | **0.02** |
|  | | Within Species | 223 | 111.192 | 0.383 |  |  |
|  | |  |  |  |  |  |  |
| Below traits | | Among Species | 33 | 389.33 | 0.448 | 6.4341 | **0.001** |
|  | | Treatment | 1 | 9.43 | 0.011 | 5.1414 | **0.004** |
|  | | Sp*Treatment | 33 | 62.35 | 0.072 | 1.0304 | 0.433 |
|  | | Within Species | 223 | 408.9 | 0.470 |  |  |
| **Analogous traits** | |  |  |  |  |  |  |
| Leaf traits | | Among Species | 33 | 361.86 | 0.624 | 15.3667 | **0.001** |
|  | | Treatment | 1 | 26.89 | 0.046 | 37.6862 | **0.001** |
|  | | Sp*Treatment | 33 | 32.12 | 0.055 | 1.3639 | **0.042** |
|  | | Within Species | 223 | 159.13 | 0.274 |  |  |
|  | |  |  |  |  |  |  |
| Root traits | | Among Species | 33 | 210.23 | 0.362 | 4.4865 | **0.001** |
|  | | Treatment | 1 | 6.72 | 0.012 | 4.7343 | **0.013** |
|  | | Sp*Treatment | 33 | 46.39 | 0.080 | 0.9901 | 0.516 |
|  | | Within Species | 223 | 316.65 | 0.546 |  |  |

**Supplementary Table 5**. PERMANOVAs considering only graminoids. Models for each trait, for the combination of aboveground traits, and for the combination of fine-root traits, considering species and treatment interaction. *Above* is the combination of traits: LA, SLA, LDMC, height. *Below* is the combination of traits: AvgD, SRL, RDMC. Analogous leaf and fine-root trait pairs: SLA, LDMC (*Leaf*), SRL, RDMC (*Root*). Significant p-values are shown in bold text (p<0.05). Trait abbreviations are explained in Supplementary Table 2.

|  |  | **Source of variation** | **Df** | **SumOfSqs** | **R2** | **F** | **p-value** |
| --- | --- | --- | --- | --- | --- | --- | --- |
| **Aboveground traits** | |  |  |  |  |  |  |
| logHeight | | Among Species | 15 | 114.151 | 0.736 | 30.9905 | **0.001** |
|  | | Treatment | 1 | 6.196 | 0.040 | 25.2301 | **0.001** |
|  | | Sp*Treatment | 15 | 4.204 | 0.027 | 1.1414 | 0.348 |
|  | | Within Species | 124 | 30.45 | 0.196 |  |  |
|  | |  |  |  |  |  |  |
| logLA | | Among Species | 15 | 125.567 | 0.810 | 37.8311 | **0.001** |
|  | | Treatment | 1 | 0.15 | 0.001 | 0.6797 | 0.419 |
|  | | Sp*Treatment | 15 | 1.844 | 0.012 | 0.5557 | 0.921 |
|  | | Within Species | 124 | 27.438 | 0.177 |  |  |
|  | |  |  |  |  |  |  |
| logLDMC | | Among Species | 15 | 49.278 | 0.318 | 3.9405 | **0.001** |
|  | | Treatment | 1 | 0.004 | 0.00003 | 0.0053 | 0.946 |
|  | | Sp*Treatment | 15 | 2.34 | 0.015 | 0.1871 | 1 |
|  | | Within Species | 124 | 103.378 | 0.667 |  |  |
|  | |  |  |  |  |  |  |
| logSLA | | Among Species | 15 | 92.269 | 0.595 | 13.3769 | **0.001** |
|  | | Treatment | 1 | 2.242 | 0.014 | 4.8753 | **0.024** |
|  | | Sp*Treatment | 15 | 3.468 | 0.022 | 0.5028 | 0.938 |
|  | | Within Species | 124 | 57.021 | 0.368 |  |  |
|  | |  |  |  |  |  |  |
| Above traits | | Among Species | 15 | 381.26 | 0.615 | 14.4388 | **0.001** |
|  | | Treatment | 1 | 8.59 | 0.014 | 4.8809 | **0.003** |
|  | | Sp*Treatment | 15 | 11.86 | 0.019 | 0.449 | 1 |
|  | | Within Species | 124 | 218.29 | 0.352 |  |  |
| **Fine-root traits** | |  |  |  |  |  |  |
| logAvgD | | Among Species | 15 | 43.368 | 0.280 | 3.723 | **0.001** |
|  | | Treatment | 1 | 5.626 | 0.036 | 7.2449 | **0.008** |
|  | | Sp*Treatment | 15 | 9.708 | 0.063 | 0.8334 | 0.626 |
|  | | Within Species | 124 | 96.297 | 0.621 |  |  |
|  | |  |  |  |  |  |  |
| logRDMC | | Among Species | 15 | 47.042 | 0.304 | 3.8229 | **0.001** |
|  | | Treatment | 1 | 0.765 | 0.005 | 0.932 | 0.326 |
|  | | Sp*Treatment | 15 | 5.471 | 0.035 | 0.4446 | 0.957 |
|  | | Within Species | 124 | 101.723 | 0.656 |  |  |
|  | |  |  |  |  |  |  |
| logSRL | | Among Species | 15 | 56.356 | 0.364 | 6.6631 | **0.001** |
|  | | Treatment | 1 | 21.207 | 0.137 | 37.6098 | **0.001** |
|  | | Sp*Treatment | 15 | 7.518 | 0.049 | 0.8888 | 0.604 |
|  | | Within Species | 124 | 69.919 | 0.451 |  |  |
|  | |  |  |  |  |  |  |
| Below traits | | Among Species | 15 | 146.77 | 0.316 | 4.5282 | **0.001** |
|  | | Treatment | 1 | 27.6 | 0.059 | 12.772 | **0.001** |
|  | | Sp*Treatment | 15 | 22.7 | 0.049 | 0.7002 | 0.902 |
|  | | Within Species | 124 | 267.94 | 0.576 |  |  |
| **Analogous traits** | |  |  |  |  |  |  |
| Leaf traits | | Among Species | 15 | 141.547 | 0.457 | 7.2951 | **0.001** |
|  | | Treatment | 1 | 2.246 | 0.007 | 1.7366 | 0.172 |
|  | | Sp*Treatment | 15 | 5.808 | 0.019 | 0.2993 | 1 |
|  | | Within Species | 124 | 160.398 | 0.517 |  |  |
|  | |  |  |  |  |  |  |
| Root traits | | Among Species | 15 | 103.398 | 0.334 | 4.9799 | **0.001** |
|  | | Treatment | 1 | 21.971 | 0.071 | 15.8728 | **0.001** |
|  | | Sp*Treatment | 15 | 12.988 | 0.042 | 0.6255 | 0.932 |
|  | | Within Species | 124 | 171.642 | 0.554 |  |  |

**Supplementary Table 6**. Linear mixed effects models for graminoids and forbs separately. Models for both treatments considering species as a random factor. Proportion of variance explained by species and within species. Log-traits were considered. Traits abbreviations are explained in Supplementary Table 2.

| **Growth form** | **Treatment** | **Trait** | **Species** | **Within Species** |
| --- | --- | --- | --- | --- |
| Graminoids | Control | Height | 0.803 | 0.197 |
|  |  | LA | 0.747 | 0.253 |
|  |  | LDMC | 0.157 | 0.843 |
|  |  | SLA | 0.492 | 0.508 |
|  |  | AvgD | 0.235 | 0.765 |
|  |  | RDMC | 0.168 | 0.832 |
|  |  | SRL | 0.328 | 0.672 |
|  | Drought | Height | 0.714 | 0.286 |
|  |  | LA | 0.824 | 0.176 |
|  |  | LDMC | 0.207 | 0.793 |
|  |  | SLA | 0.619 | 0.381 |
|  |  | AvgD | 0.164 | 0.836 |
|  |  | RDMC | 0.218 | 0.782 |
|  |  | SRL | 0.387 | 0.613 |
| Forbs | Control | Height | 0.768 | 0.232 |
|  |  | LA | 0.859 | 0.141 |
|  |  | LDMC | 0.610 | 0.390 |
|  |  | SLA | 0.677 | 0.323 |
|  |  | AvgD | 0.650 | 0.350 |
|  |  | RDMC | 0.071 | 0.929 |
|  |  | SRL | 0.538 | 0.462 |
|  | Drought | Height | 0.786 | 0.214 |
|  |  | LA | 0.866 | 0.134 |
|  |  | LDMC | 0.573 | 0.427 |
|  |  | SLA | 0.660 | 0.340 |
|  |  | AvgD | 0.583 | 0.417 |
|  |  | RDMC | 0.073 | 0.927 |
|  |  | SRL | 0.529 | 0.471 |

**Supplementary Table 7**. Linear mixed effects models for graminoids and forbs separately. Models for each single trait considering species and treatment as random factors. Proportion of variance explained by species, treatment, and within species. Log-traits were considered. Traits abbreviations are explained in Supplementary Table 2.

| **Growth form** | **Trait** | **Species** | **Treatment** | **Within Species** |
| --- | --- | --- | --- | --- |
| Graminoids | Height | 0.740 | 0.026 | 0.234 |
|  | LA | 0.778 | 3.01E-09 | 0.222 |
|  | LDMC | 0.171 | 1.25E-09 | 0.829 |
|  | SLA | 0.544 | 6.44E-09 | 0.456 |
|  | SRL | 0.282 | 0.215 | 0.503 |
|  | AvgD | 0.198 | 0.047 | 0.755 |
|  | RDMC | 0.183 | 1.92E-09 | 0.817 |
| Forbs | Height | 0.773 | 2.99E-09 | 0.227 |
|  | LA | 0.861 | 6.99E-09 | 0.139 |
|  | LDMC | 0.579 | 0.019 | 0.401 |
|  | SLA | 0.578 | 0.136 | 0.286 |
|  | SRL | 0.501 | 2.98E-09 | 0.499 |
|  | AvgD | 0.615 | 1.49E-09 | 0.385 |
|  | RDMC | 0.070 | 0.028 | 0.903 |


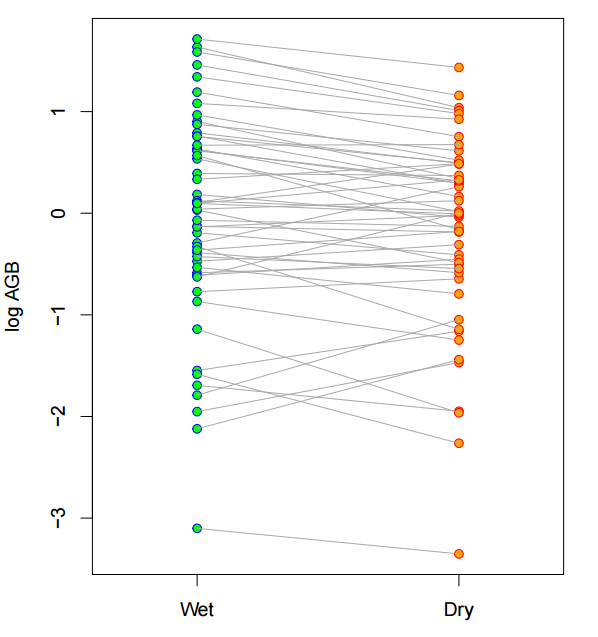


**Supplementary Figure 1**. Reductions in plant aboveground biomass for most species under drought conditions. 35 species out of 52 had a reduction in aboveground biomass (AGB) under the drought treatment. Each point represents the mean aboveground biomass of each species under wet (control) and dry (drought) conditions. Log-AGB was considered.
